# Reanalysis of the Multi‐Biomarker Disease Activity Score for Assessing Disease Activity in the Abatacept Versus Adalimumab Comparison in Biologic‐Naive Rheumatoid Arthritis Subjects with Background Methotrexate Study: Comment on the Article by Fleischmann et al

**DOI:** 10.1002/art.39981

**Published:** 2017-03-03

**Authors:** Jeffrey R. Curtis, Grace C. Wright, Vibeke Strand, Charles S. Davis, Elena Hitraya, Eric H. Sasso

**Affiliations:** ^1^ University of Alabama at Birmingham; ^2^ New York University Langone Medical Center New York NY; ^3^ Stanford University School of Medicine Stanford CA; ^4^ CSD Biostatistics, Inc. Oro Valley AZ; ^5^ Crescendo Bioscience South San Francisco CA

To the Editor:

We read with great interest the report by Fleischmann et al 1, which presents post hoc analyses of multi‐biomarker disease activity (MBDA) scores measured using serum samples from AMPLE (Abatacept versus Adalimumab Comparison in Biologic‐Naive Rheumatoid Arthritis Subjects with Background Methotrexate), a study sponsored by Bristol‐Myers Squibb that compared abatacept versus adalimumab in rheumatoid arthritis (RA) patients with inadequate response to methotrexate 2. The article reported 3 main results: 1) in the first year, mean MBDA scores decreased significantly less with abatacept treatment than with adalimumab, yet clinical responses by Disease Activity Score in 28 joints using the C‐reactive protein level (DAS28‐CRP) 3 were similar between the 2 treatment groups; 2) RA disease activity category (i.e., low, moderate, or high) as classified by the MBDA score was often discordant with the classification according to the DAS28‐CRP, Clinical Disease Activity Index (CDAI) 4, Simplified Disease Activity Index (SDAI) 5, or Routine Assessment of Patient Index Data 3 6; and 3) radiographic data were interpreted as demonstrating that MBDA scores were not associated with radiographic progression. Based on these 3 results, it was concluded that the MBDA score should not be used to guide decision‐making in the management of RA. We wish to demonstrate limitations of these analyses that raise questions regarding the interpretation of these 3 results, as well as the overall conclusion that was reached.

Addressing the 3 results in reverse order, we note that the relationship between radiographic progression and MBDA scores was assessed using a method that seems inadequate to test the desired hypothesis, that the MBDA score is associated with radiographic nonprogression. This analysis, presented in Figure 2D of the article, seemingly shows the proportion of patients whose disease did not progress radiographically within each MBDA category. In other words, one would expect the denominator of this proportion to be the number of patients within each MBDA category, and the numerator to be the number of nonprogressors in that category. However, upon careful inspection, this is not what the figure shows. Instead, Figure 2D describes the proportions of patients who were nonprogressors, classified as having low, moderate, or high disease activity by MBDA score. This approach is not informative about the relationship between MBDA scores and radiographic progression because the same denominator was used for each MBDA category. Thus, the proportions reflect only the distribution of MBDA scores among the nonprogressors. A more conventional analysis, such as that described below, or cumulative probability plots showing changes in modified total Sharp scores 7 by MBDA category, would be more informative to determine the likelihood of radiographic progression, conditional on patient MBDA category.

To this point, we reanalyzed the year 1 radiographic outcomes using the data provided in the original publication 1 to determine if the percentage of radiographic progressors increased with increasing MBDA scores, using previously published methods 8. We computed the proportion of radiographic progressors in each MBDA category by dividing the number of progressors in each MBDA category by the number of patients in that category 1. However, the exact numbers of patients with missing data (overall 8 of 189 in the abatacept arm and 4 of 190 in the adalimumab arm), and of patients with radiographic progression (overall 19 of 189 and 21 of 190, respectively), were not provided for the individual MBDA categories. Therefore, to establish the boundaries on all possible results that would be compatible with the data provided, we conducted a sensitivity analysis that varied the distributions of progressors and patients with missing data across MBDA categories. At one extreme (Scenario 1, least conservative), all progressors were assigned to the highest possible MBDA categories. At the other extreme (Scenario 2, most conservative), all progressors were assigned to the lowest possible MBDA categories (Supplementary Tables 1 and 2, on the *Arthritis & Rheumatology* web site at http://onlinelibrary.wiley.com/doi/10.1002/art.39981/abstract).

The results of the above‐described reanalysis are shown in Figure 1. In Scenario 1, there was a strong and statistically significant association between MBDA category and radiographic progression in both the abatacept and adalimumab arms. At the other extreme, in Scenario 2, the adalimumab results were statistically significant and there was a similar, albeit nonsignificant, trend in the abatacept arm (*P* = 0.068). Given that the actual result must lie at or between the extremes of Scenarios 1 and 2 in this sensitivity analysis, the results as presented in Figure 2D of the article by Fleischmann et al do not support the conclusion that MBDA scores did not reflect radiographic progression status in the AMPLE trial 1. Rather, our reanalysis indicates that the MBDA category is positively associated with radiographic progression in the AMPLE study, as has been reported in other RA cohorts 9, 10, 11, 12, 13. We invite replication of this reanalysis using patient‐level data.

**Figure 1:**
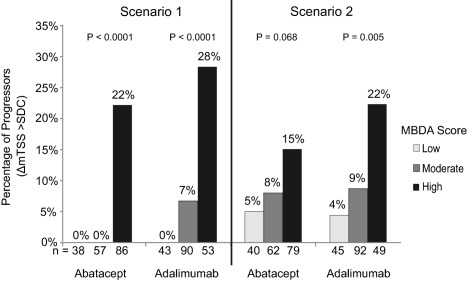
Sensitivity analysis examining the association between radiographic progression and multi‐biomarker disease activity (MBDA) score in the AMPLE study (Abatacept versus Adalimumab Comparison in Biologic‐Naive Rheumatoid Arthritis Subjects with Background Methotrexate). Percentages of patients with radiographic progression at year 1 were analyzed by year 1 MBDA category for the abatacept arm and adalimumab arm, using published data from Fleischmann et al 1. Progressors were defined as having changes in the modified total Sharp score (ΔmTSS) exceeding the smallest detectable change (SDC) 1. The number of progressors in an individual MBDA category (low, moderate, or high) was determined in 2 steps. First, the total number of progressors in the 3 MBDA categories was calculated by subtracting the total number of nonprogressors, summed from Figure 2D in the report by Fleischmann and colleagues, from the total number of patients, obtained from Supplementary Table 5 in their report 1. Next, the total number of progressors was distributed across the 3 MBDA categories in 2 scenarios, representing the most extreme possibilities compatible with the reported data: Scenario 1 (least conservative) assigned all progressors to the highest possible MBDA categories; Scenario 2 (most conservative) assigned all progressors to the lowest possible MBDA categories (for numerical details, see Supplementary Tables 1 and 2, on the *Arthritis & Rheumatology* web site at http://onlinelibrary.wiley.com/doi/10.1002/art.39981/abstract). N values on the x‐axis are the number of patients in the MBDA category, i.e., the sum of progressors (P) and nonprogressors (NP) in that MBDA category. The percentage of patients with radiographic progression in each MBDA category is 100 × (P/[P + NP]). Statistical significance was determined by Mantel‐Haenszel test for trend, assuming ordinality in the MBDA categories.

Second, the MBDA score was initially developed to correlate with the DAS28‐CRP, and its RA disease activity categories were established using cutoffs that are specific to the DAS28‐CRP 14. Thresholds for DAS28‐CRP RA disease activity categories are systematically lower than the corresponding cutoffs in the DAS28 using the erythrocyte sedimentation rate (ESR) 15, 16, as noted in the editorial accompanying Fleischmann and colleagues' article 17. The comparison of MBDA scores versus DAS28‐CRP in the article used the DAS28‐ESR category thresholds, and therefore yielded more discordance than would have been expected between MBDA categories and DAS28‐CRP categories using DAS28‐CRP thresholds. For both the DAS28‐CRP and the other clinical measures examined (e.g., CDAI, SDAI), the claim of no clear association between MBDA scores and commonly used, validated clinical measures was not supported by a statistical test of no association that cross‐classified the MBDA category of each patient with his or her clinically defined RA disease activity category.

Regardless, because the MBDA score was designed to complement, not supplant, clinical assessment, some discordance between clinical and laboratory‐based assessments is not only expected 14, 18, 19, but desirable. Otherwise, the laboratory test would contain no incremental information beyond that offered by clinical evaluation. Several studies have shown that high MBDA scores were associated with radiographic progression and low scores with nonprogression 9, 10, 11, 12, 13, even when the patient was classified as having low disease activity or remission based on clinical measures (e.g., the DAS28‐CRP) 9, 12, 20.

The third and last result reported by Fleischmann et al that we wish to address is that the mean decrease in MBDA score was larger for adalimumab‐treated patients compared with abatacept‐treated patients at month 3 (day 85) and beyond. The approximate difference between the 2 arms was 3–4 units at month 3, 4–5 units at year 1, and 0–1 units at year 2; these differences were statistically significant at month 3 and year 1 but not year 2. While these differences may appear large in Figure 1 of the article, where the y‐axis scale ranged from +2 to −16 units, the MBDA score is measured on a 1–100 scale. More importantly, the difference between the 2 treatment arms at month 3 and year 1 is approximately equal to the measurement error of the MBDA score (4.5 units) 21. As was pointed out in the report 1, this small difference in the mean MBDA scores between the 2 treatment arms is likely clinically irrelevant.

For unclear reasons, data on approximately one‐fifth of patients were missing from the AMPLE MBDA analysis, such that a systematic bias as to why they were not analyzed cannot be excluded. We also note that 31% of patients had missing MBDA data at year 2 compared with year 1, which exceeds the 8.5% decline in total patient numbers from the end of year 1 to the end of year 2 in the overall AMPLE trial 2, 22.

In summary, the analysis by Fleischmann et al has several limitations that raise uncertainties about the interpretations presented in the article. We note that the authors reached the conclusion that the MBDA score is not useful for RA patient management based on interpretations that questioned the validity of the MBDA test in this one study, without considering all the available evidence. We would encourage readers to make the distinction between the scientific validity of a diagnostic test and its clinical utility. There is already a sizable evidence base supporting the development and validation of the MBDA test in diverse RA patient cohorts 9, 10, 11, 12, 13, 18, 19, 20, 23, 24, 25. A prospective clinical trial is underway to rigorously evaluate its clinical utility and its potential role in RA patient management 26.

*Dr. Curtis has received consulting fees, speaking fees, and/or honoraria from Crescendo Bioscience, Inc., Pfizer, and Bristol‐Myers Squibb (less than $10,000 each) and from UCB, Amgen, Janssen, and the CORRONA registry (more than $10,000 each). Dr. Wright has received consulting fees, speaking fees, and/or honoraria from Medac Pharma (less than $10,000) and from AbbVie, Bristol‐Myers Squibb, and Crescendo Bioscience, Inc. (more than $10,000 each). Dr. Strand has received consulting fees, speaking fees, and/or honoraria from AbbVie, Alder, Amgen, Bristol‐Myers Squibb, Boehringer Ingelheim, Celgene, Celltrion, the CORRONA registry, Crescendo Bioscience, Inc., GlaxoSmithKline, Janssen, Lilly, Merck, Novartis, Pfizer, Protagen, Regeneron, Samsung, Sandoz, Sanofi, and UCB (less than $10,000 each). Dr. Davis has received consulting fees from Crescendo Bioscience, Inc. (less than $10,000). Drs. Hitraya and Sasso are shareholders of Myriad Genetics, Inc*.


Jeffrey R. Curtis, MD, MS, MPH
*University of Alabama at Birmingham*
Grace C. Wright, MD, PhD
*New York University Langone Medical Center*
*New York, NY*
Vibeke Strand, MD
*Stanford University School of Medicine*
*Stanford, CA*
Charles S. Davis, PhD
*CSD Biostatistics, Inc*.
*Oro Valley, AZ*
Elena Hitraya, MD, PhD
Eric H. Sasso, MD
*Crescendo Bioscience*
*South San Francisco, CA*

## Supporting information


**Supplementary Table 1**. Abatacept at 1 year: Sensitivity analyses showing the distributions of data describing the least and most conservative scenarios for the association between the MBDA score and radiographic progression
**Supplementary Table 2**. Adalimumab at 1 year: Sensitivity analyses showing the distributions of data describing the least and most conservative scenarios for the association between the MBDA score and radiographic progression
