# Supplementary material for: Reanalysis of the Multi‐Biomarker Disease Activity Score for Assessing Disease Activity in the Abatacept Versus Adalimumab Comparison in Biologic‐Naive Rheumatoid Arthritis Subjects with Background Methotrexate Study: Comment on the Article by Fleischmann et al
Source: Arthritis Rheumatol. 2017 Mar 3;69(4):863–5. doi: 10.1002/art.39981 (PMC6749941; doi:10.1002/art.39981)
Supplement: Supplementary file 1 — Supplementary Table 1. Abatacept at 1 year: Sensitivity analyses showing the distributions of data describing the least and most conservative scenarios for the association between the MBDA score and radiographic progression Supplementary Table 2. Adalimumab at 1 year: Sensitivity analyses showing the distributions of data describing the least and most conservative scenarios for the association between the MBDA score and radiographic progression [file ART-69-863-s001.docx]

**Supplementary Tables 1 and 2**

**Supplementary Table 1.** Abatacept at 1 year: Sensitivity analyses showing the distributions of data describing the least and most conservative scenarios for the association between the MBDA score and radiographic progression

| **Source of data** | **Patient group** | **MBDA score category** | | | **Total** |
| --- | --- | --- | --- | --- | --- |
|  |  | **Low** | **Moderate** | **High** |  |
| Figure 2D* | NP | 38 | 57 | 67 | 162 |
| Supplementary Table 5* | P + NP | NA | NA | NA | 181 |
| Table 1* | P + NP + missing data | 40 | 62 | 87 | 189 |
| Calculated | P + missing data | 2 | 5 | 20 | 27 |
| Scenario 1: Least conservative ** | P | 0 | 0 | 19 | 19 |
|  | Missing data | 2 | 5 | 1 | 8 |
|  | P / (P + NP) | 0/38 | 0/57 | 19/86 | 19/181 |
|  | % progressors | **0%** | **0%** | **22.1%** | 10.5% |
| Scenario 2: Most conservative ** | P | 2 | 5 | 12 | 19 |
|  | Missing data | 0 | 0 | 8 | 8 |
|  | P / (P + NP) | 2/40 | 5/62 | 12/79 | 19/181 |
|  | % progressors | **5.0%** | **8.1%** | **15.2%** | 10.5% |

NA = not available; NP = radiographic non-progressors; P = radiographic progressors

* Fleischmann R, Connolly SE, Maldonado MA, Schiff M. Estimating disease activity using multi-biomarker disease activity scores in patients with rheumatoid arthritis treated with abatacept or adalimumab. Arthritis Rheumatol 2016. doi: 10.1002/art.39714.

**For methodology used to obtain values in each scenario, see legend of Figure 1.

**Supplementary Table 2.** Adalimumab at 1 year: Sensitivity analyses showing the distributions of data describing the least and most conservative scenarios for the association between the MBDA score and radiographic progression

| **Source of data** | **Patient group** | **MBDA score category** | | | **Total** |
| --- | --- | --- | --- | --- | --- |
|  |  | **Low** | **Moderate** | **High** |  |
| Figure 2D* | NP | 43 | 84 | 38 | 165 |
| Supplementary Table 5* | P + NP | NA | NA | NA | 186 |
| Table 1* | P + NP + missing data | 45 | 92 | 53 | 190 |
| Calculated | P + missing data | 2 | 8 | 15 | 25 |
| Scenario 1: Least conservative** | P | 0 | 6 | 15 | 21 |
|  | Missing data | 2 | 2 | 0 | 4 |
|  | P / (P + NP) | 0/43 | 6/90 | 15/53 | 21/186 |
|  | % progressors | **0%** | **6.7%** | **28.3%** | 11.3% |
| Scenario 2: Most conservative** | P | 2 | 8 | 11 | 21 |
|  | Missing data | 0 | 0 | 4 | 4 |
|  | P / (P + NP) | 2/45 | 8/92 | 11/49 | 21/186 |
|  | % progressors | **4.4%** | **8.7%** | **22.4%** | 11.3% |

NA = not available; NP = radiographic non-progressors; P = radiographic progressors

*Fleischmann R, Connolly SE, Maldonado MA, Schiff M. Estimating disease activity using multi-biomarker disease activity scores in patients with rheumatoid arthritis treated with abatacept or adalimumab. Arthritis Rheumatol 2016. doi: 10.1002/art.39714.

**For methodology used to obtain values in each scenario, see legend of Figure 1.
